# Ginger, a Possible Candidate for the Treatment of Dementias?

**DOI:** 10.3390/molecules26185700

**Published:** 2021-09-21

**Authors:** Giovanni Schepici, Valentina Contestabile, Andrea Valeri, Emanuela Mazzon

**Affiliations:** IRCCS Centro Neurolesi “Bonino-Pulejo”, Via Provinciale Palermo, Contrada Casazza, 98124 Messina, Italy; giovanni.schepici@irccsme.it (G.S.); valentina.contestabile@irccsme.it (V.C.); andrea.valeri@irccsme.it (A.V.)

**Keywords:** ginger, dementia, neurodegeneration, neuroinflammation, Alzheimer’s disease, vascular dementia, neuroprotection

## Abstract

As the human life expectancy increases, age-linked diseases have become more and more frequent. The worldwide increment of dementia cases demands medical solutions, but the current available drugs do not meet all the expectations. Recently the attention of the scientific community was attracted by natural compounds, used in ancient medicine, known for their beneficial effects and high tolerability. This review is focused on Ginger (*Zingiber officinale*) and explore its properties against Alzheimer’s Disease and Vascular Dementia, two of the most common and devastating forms of dementia. This work resumes the beneficial effects of Ginger compounds, tested in computational in vitro and in vivo models of Alzheimer’s Disease and Vascular Dementia, along with some human tests. All these evidences suggest a potential role of the compounds of ginger not only in the treatment of the disease, but also in its prevention.

## 1. Introduction

Dementia is a disease characterized by a progressive cognitive decline linked to age that involves a reduction in personal autonomy. In general, dementia is characterized by loss of memory, problem-solving impairment, sometimes difficulty in language uttering and, more in general, a significant reduction in thinking processes [1]. Dementia affects approximately 50 million people worldwide with an increase of approximately 82 million affected patients in 2030 and in 152 million by 2050. The Global Dementia Observatory Reference Guide estimated that around 9.9 million people develop dementia each year, which equals a new case every three seconds. The clinical criteria necessary for the diagnosis of dementia or “major neurocognitive disorder”, according to the guidelines of the Diagnostic and Statistical Manual of Mental Disorders (DSM-5), are the presence of significant cognitive decline in at least one of the domains: memory, attention, language, motor programming, perception of objects, space-time perception, and executive functions [2]. The main pathogenetic hallmark underlying dementia in the elderly is neurodegeneration. The most common forms of degenerative dementia are Alzheimer’s Disease (AD), Lewy Body Dementia, Vascular Dementia (VD), Frontotemporal Lobar Degeneration, and Parkinson’s Disease. There is a group of dementias that is important to mention because of their non-neurodegenerative nature and for characteristic of being reversible with treatments. Mild cognitive forms of non-neurodegenerative dementia can be caused by vitamin deficiencies, hypothyroidism, normal pressure hydrocephalus, chronic alcohol abuse, chemotherapy, infections, intracranial formations, traumatic brain injury, and psychiatric illness [3]. Many dementia patients also show behavioural and psychological symptoms, including aggression, agitation, and depression [4].

Apart from neurogeneration, AD is characterized by senile plaques and neurofibrillary tangles (NFTs). Senile plaques are mainly composed by aggregates of β-amyloid (Aβ) peptides, while hyperphosphorylation of protein Tau causes NFTs. The resulting inflammatory state and oxidative stress cause neuronal death, which leads to cognitive impairments. Neurotransmission is consequently affected, with a significant impairment of cholinergic pathways [5].

VD refers to impaired cognitive function secondary to brain damage due to cerebral vascular disease. The damage resulting from vascular alterations is due to hypoperfusion, oxidative stress and inflammatory process, with consequent endothelial damage, rupture of the blood-brain-barrier (BBB), and activation of innate immunity [6]. Vascular lesions that lead to cognitive impairment can be cortical ischemic events or lacunar infarcts both at the cortical and subcortical levels [7].

Nowadays there are several treatments for dementias capable of temporarily improving the symptoms. However, the side effects and poor efficacy demand the discovery and design of new compounds to prevent and treat the pathology. Therefore, research today is focusing attention on medical plants such as Ginger (*Zingiber officinale*) known for centuries for their numerous beneficial properties, which could be useful for prevention and treatment of dementias. The purpose of our review is to provide an overview of the neuroprotective effects of ginger as possible supplement to current dementia therapies.

## 2. Methodology

**Figure 1:**
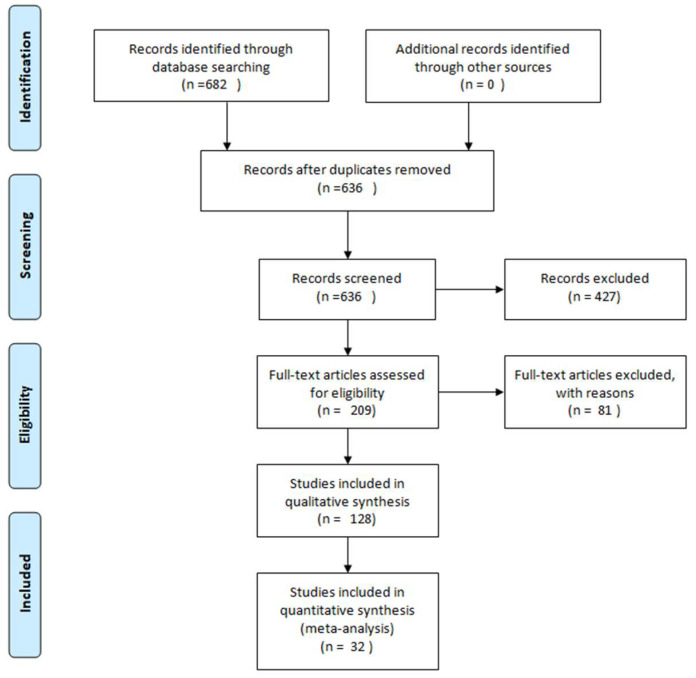
Methodology description of articles selected for this review using Prisma flow diagram [8].

We selected the publications from 2003 to 2021. The search was performed using the database PubMed Central and the keywords “ginger” and “dementia”. We considered the articles that reported the neuroprotective effects of ginger and its compounds as potential therapeutic agents in the two major forms of dementias, AD and VD. We included in our analyses the articles that reported experimental studies, excluding in such way reviews and articles not relevant, as shown in the Prisma flow diagram (Figure 1).

## 3. Ginger: Chemical Structure, Properties, and Therapeutic Potential

Ginger (*Zingiber officinale*) is a monocotyledon belonging to the Zingiberaceae family and its tuberous rhizomes are widely known for its beneficial effects as anti-inflammatory, antioxidant, anti-apoptotic, antiviral, and antibacterial [9,10]. Based on its effects, ginger could be used for the treatment of gastrointestinal disorders including nausea, vomiting, diarrhea, as well as neurological disorders, metabolic, and cardiovascular diseases [9,11].

**Figure 2:**
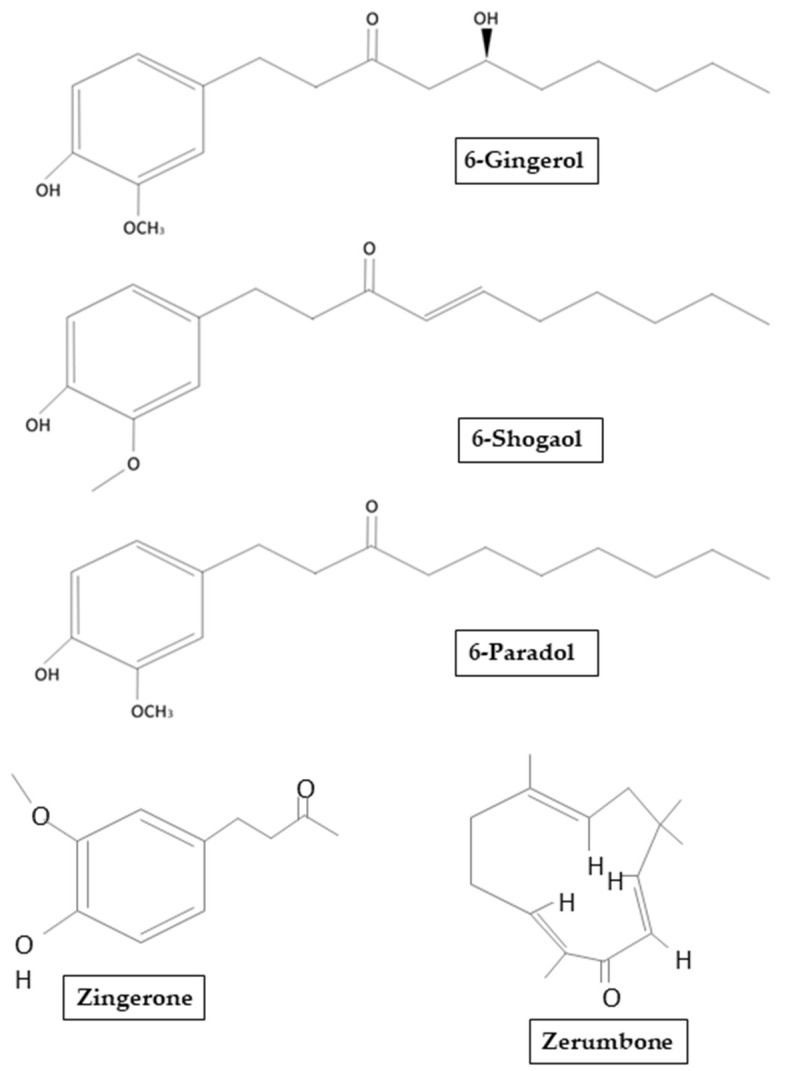
Chemical structure of main ginger derivates that will be mentioned in our review.

About 400 different components such as carbohydrates, lipids, terpenes and phenolic have been identified in ginger. These compounds have been divided into pungent and aromatic:gingerols, shogaols, zingerones, gingerdione, paradols, zerumbone and capsaicin belongs to the pungent compounds (Figure 2), while pinene, borneol, cumene, camphene, zingiberol, and bisabolen are classified as aromatic [12].

Among the biologically active compounds of terpenes, gingerols are the most abundant [13], with 6-gingerols containing a high amount [14]: analytical analyses determined that the amount of 6-gingerols was between 57.67 and 62.64 mg/g of rhizome-methanol extract [15]. Gingerols are thermally labile at high temperatures and easily subject to dehydration to form shogaol. Additionally, it has also been reported that conversion of gingerols to shogaol induced by temperatures can be affected by the type of heat (dry or wet) and also by the type of ginger (fresh or dried) [16]. Another compound obtained by the degradation of gingerols is zingerone [4-(4-hydroxy-3-methoxyphenyl)-butan-2-one]. Zingerone is obtained after cooking or drying the rhizome, it can be chemically related to aromatic compounds such as vanillin and eugenol. Usually, the biologically active compounds of ginger have been found in essential oils. It has been demonstrated that after steam extraction, ginger contains a small percentage of essential oil rich in sesquiterpene hydrocarbons such as zingiberene and also small amounts of monoterpenic hydrocarbons and oxygenated compounds. However, attention must be paid when selecting the drying method or the freshness of the ginger, because these can affect the chemical structure and so alter the efficacy of the resulting compound [13]. Indeed, the main sesquiterpene hydrocarbon compounds in ginger oil obtained from fresh rhizome were α-zingiberene (27–30%), α-curcumene (8–9%), β-sesquifellandrene (4.8%), and bisabolen (3.2%). Instead, in a lesser amount, ginger essential oil contains acyclic α-farnesene and a variety of monoterpenic compounds including limonene, myrcene, α-pinene, borneol, citronellol, geraniol, and linalool [17]. Paradols, another group of pungent compounds, is known for exhibiting antioxidant and anti-inflammatory properties, in particular exerting action against Cyclooxygenase 1 (COX-1) [18].

Although the deterioration of ginger root can lead to the formation of mycotoxins such as mycophenolic acid, at present no onset of disease in humans has been shown [19]. There are few data on the toxicity of ginger following its ingestion. It has been reported that the daily consumption of fresh ginger is between 2 and 4 g or 0.5 and 1.0 g for dry rhizome powder. Noteworthy that in a clinical study of coronary heart disease patients, it was demonstrated that the administration of powdered ginger in a single dose of 10 g did not lead to adverse events [20].

In regard to the bioavailability of ginger and its compounds, it has been shown in rats that gingerol undergoes conjugation and oxidation in the phenolic side chain. About 48% of 6-gingerol, orally administered for over 60 h, has been found in the bile of these animals. Approximately 12% as metabolites of 6-gingerol (vanillic acid and ferulic acid) has been observed in the urine [21]. Therefore, based on its beneficial effects shown, as well as the poor neurotoxicity, ginger could be potentially used as a supplement for the prevention and treatment of dementias.

## 4. Alzheimer’s Disease

AD is the main form of dementia which can lead to progressive cognitive deficits and can be classified into sporadic late onset AD (SAD) and familial early onset AD (FAD), with an higher prevalence of the sporadic form [22]. Several factors can contribute to the disease, including aging, mutation on genes such as the apolipoprotein E (APOE) ε4 gene, Presenilin 1 (PSEN 1) and Presenilin 2 (PSEN 2) as well as risk factors such as obesity and hypertension. Oxidative stress, apoptosis, and inflammatory responses are involved in the incidence of AD [9]. The disease is mainly characterized by senile plaques, in which fibrillar Aβ is the major component [23], as well as of NFTs formation by hyperphosphorylated Tau protein. Aβ peptides originates from the aberrant proteolysis of the amyloid precursor protein (APP), a trans-membrane protein involved in brain homeostasis, by the enzyme γ-secretase [24]. Aβ peptide aggregates can induce neuroinflammation, apoptosis and compromise the synaptic plasticity thus leading to cerebral neurodegeneration [25]. Tau, in physiological condition, plays an important role in axonal transport through the microtubule stabilization [26] and its correct functioning is regulated by the perfect balance between its phosphorylation and dephosphorylation [27]. Glycogen synthase kinase-3 (GSK-3) mediate Tau hyperphosphorylation [28,29] and aggregates of Aβ can facilitate GSK-3 activity, suggesting a reinforced mechanism between the two main hallmarks of AD [30].

The association between weight gain could be one of the risk factors of developing dementia over the years. Among the main complications of obesity are insulin resistance and consequent hyperinsulinism, which could play a role in the clearance of Aβ from the brain and thus increase the risk of dementia and AD. Adipose secretes proteins and hormones that develop inflammation in the brain tissue in response to high levels of insulin. Terzi et al. [31] highlight the role of insulin-resistance as one of the key factors of brain alterations in AD patients. In the brain, insulin is involved in the maintenance of cognitive functions, since elevated level of insulin were found in regions of the brain deputy for memory and learning. Insulin-resistance condition is correlated with increased expression of GSK3-β and Aβ peptide, which shares with insulin the degradation enzyme, so hyperinsulinemia favours both NFTs and Aβ plaques formation. As a result, both diabetic patients and obese people have increased risk of dementia and cognitive impairment.

Nowadays, limited approved therapies are available for dementia, thus natural compounds such as ginger could be useful for the treatment of AD [32]. The beneficial effects of ginger and its compounds have been demonstrated in experimental studies and also in human [33,34]. Ginger constituents and in particularly gingerols have been known to have pharmacological properties that mimic the non-steroidal anti-inflammatory drugs (NSAIDs) double action, but with less side effects [35,36,37]. Consequently, ginger can exert a dual action by inhibiting the activity of enzymes implicated in inflammatory processes, such as COX-2 and 5-lipoxygenase (5-LOX) involved in the biosynthesis of leukotrienes (LTs) [35,38].

## 5. Effects of Ginger in AD Studies

Several studies have shown the therapeutic potential of ginger and its constituents for the improvement of cognitive deficits thanks to their anti-amyloidogenic potential, cholinesterase inhibition, and neuroprotective properties [39,40].

Nowadays, there are only a few drugs approved for the treatment of AD. Therefore, limited therapeutic targets and several side effects have prompted researchers to investigate multi-target drugs to achieve safer and broader therapeutic potential. Based on the beneficial properties of ginger and its components, several studies for the development of new AD drugs have been performed.

### 5.1. Computational Studies

Azam et al. [41] through molecular docking, a computational method useful to designing AD drugs by receptor ligand binding investigated the interactions of 12 ginger compounds with multi-molecular targets, as well as the possible mechanisms related to AD pathology. In order to design anti-AD drugs, the interactions of ginger compounds including gingerol, shogaol, and zingerone with multi-molecular targets were shown, as well as the possible mechanisms related to AD pathology. The study results identified several potential protein targets or enzymes involved in AD, including Tumor necrosis factor-α (TNF-α) converting enzyme (TACE), AChE, BChE, NOS, COX-1, COX-2, c-jun N-terminal kinase (JNK), and N-methyl-D-aspartate (NMDA). AChE is the most promising molecular target, based on the analysis of binding energy and ligand/target receptor interactions, and JNK represent the least choice. As verified in experimental studies, the molecular docking showed that butyrylcholinesterase (BChE), TACE, COX-2, NOS, and NMDA, were identified as the best putative targets for ginger bioactive compounds.

**Figure 3:**
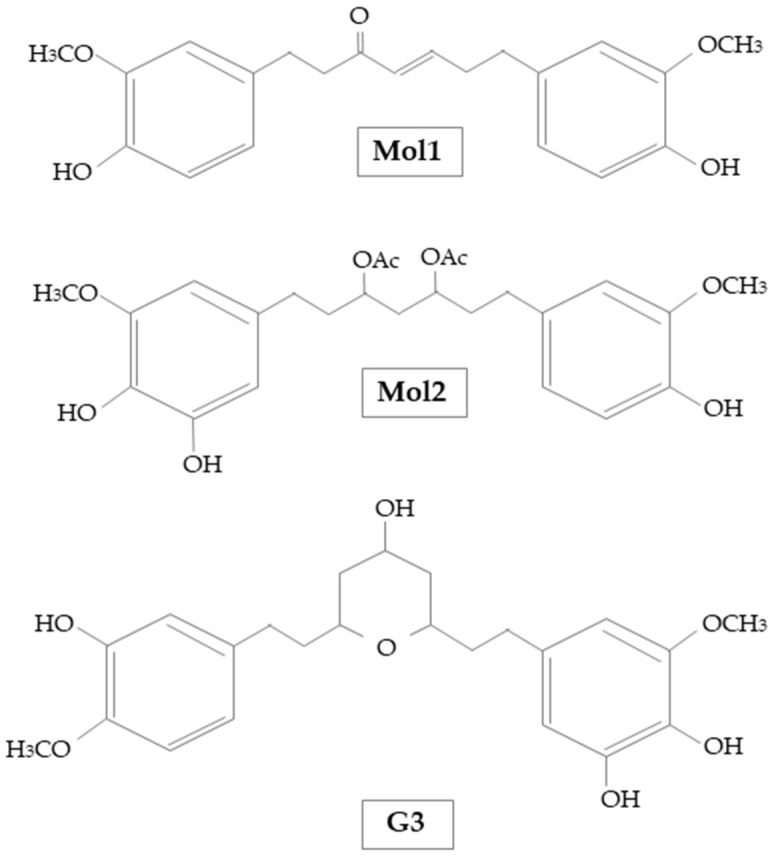
Chemical structure of Mol1, Mol2, and G3 used by Cuya et al. [42,43].

Cuya et al. [42] demonstrated the AChE inhibitory effects of some active components of ginger extracts by comparing them to donepezil an AChE inhibitor drug used to treat AD. The molecular docking study results reported that [(E)-1,7-bis(4-hydroxy-3-methoxyphenyl)hept-4-en-3-on] (Mol1) and [1-(3,4-dihydroxy-5-methoxyphenyl)-7-(4-hydroxy-3-ethoxyphenyl) heptane-3,5-diyl diacetate] (Mol2), could be effective as natural AChE inhibitors and their action is not different from donepezil. Moreover, it has been shown that ginger compounds inhibited AChE activity by different binding sites, in particular the choline binding pocket (Trp86). Indeed, all ginger compounds showed hydrophobic interactions with Trp86. Similarly, it has been observed that it is necessary to avoid the repulsive interaction with Glu202 to design AChE inhibitors [42]. Additionally, Cuya et al. [43] investigated the effects of ginger compounds on BChE compared with donepezil. The results of the studies reported that (E)-1.7-bis(4-hydroxy-3-methoxyphenyl)hept-4-en-3-one and 5-[(2S,4R,6R)-4-hydroxy-6-[2-(4-methoxyphenyl)ethyl]oxan-2-yl]-3-methoxybenzene-1,2-diol (G3), could be useful as potential inhibitors of BChE, effective as donepezil (Figure 3). Moreover, it was observed that ginger compounds inhibited the activity of BChE by binding Trp82 and Tyr332 residues. Likewise, it has been found that repulsive interactions with Glu197 needs to be avoided for the design of BChE inhibitory compounds.

The potential effect of zerumbone as a cholinesterase inhibitor was evaluated by Hwang et al, in vitro by enzyme assays, in silico docking, and simulation of absorption, distribution, metabolism, excretion, and toxicity. The results of the study demonstrated that zerumbone inhibited AChE and BChE, assessed by an anticholinesterase activity assay and measured by zerumbone kinetics. Moreover, it has been shown through computational docking that zerumbone inhibited the AChE activity by different binding sites including TYR72, ASP74, LEU76, TYR124, TRP286, PHE297, TYR337, and TYR341. Zerumbone also inhibited the BChE activity using the binding sites ASP70, TRP82, PHE329, TYR332, TRP430, and HIS438. Furthermore, thanks to its high permeability through the BBB, zerumbone has been suggested for the role of inhibitor both of AChE and BChE. Overall, the computational studies have shown a potential efficacy of ginger in the prevention of AD [44].

Aguiar-Furucho et al. [45] demonstrated the beneficial effects on the consumption of terpenoid-based nutrients, such as ginger, and foods containing fisetin, such as strawberries, tomatoes, oranges, and cucumbers. To investigate the AD symptoms, the authors used a computational model of koniocortex, a region of cerebral cortex related to the processing of sensory information. The experimental model allows to analyse the memory deficits induced by synaptic pruning, modifying the levels of acetylcholine and GABA-A signalling. In particular, it has been shown that GABA-A, can prevent Aβ-induced neuronal death through the inhibition of the excitatory action of glutamate. GABA-A receptor impairment has been reported in AD, due to impaired interactions between GABA-A receptor and benzodiazepine. This can explain the dysregulation of neuronal excitability and the reduction of sensory stimuli as the early symptoms of AD. The results obtained showed that terpenoids, such as ginger and fisetin, contribute to the expression of GABA-A receptors and thus improve cognitive function. Hence, phytocompounds such as ginger have proven to be potential therapeutic tools for AD.

### 5.2. In Vivo and In Vitro Studies

The neuroprotective effects of ginger on SAD have been investigated in vivo by El Halawany et al. [46]. The authors model the disease in mice by a single intracerebroventricular injection of streptozotocin (3 mg/kg) and 6-gingerol was administrated intraperitoneally. Celecoxib is a COX-2 inhibitor and was used as control (30 mg/kg). The scores in Morris Water Maze and Y-maze tests demonstrated a significant improvement in the 6-gingerol-treated group. Moreover, 6-gingerol and celecoxib treated mice showed a reduction of Aβ_42_ levels, as well as β-secretase, Gamma-secretase subunit APH-1A (APH1a) and COX-2, with an increased in α-secretase activity. In conclusion, the study has demonstrated the neuroprotective effects of 6-gingerol and its action in repress neuroinflammation and amyloidogenesis, which preserved the animals from behavioural and memory deficits.

Since the disease progression is characterized by a loss of cholinergic neurons, most AD drug therapies have focused the attention on the cholinergic system. Therefore, therapeutic approaches may involve the muscarinic receptors or inhibition of Acetylcholinesterase (AChE) to increase the availability of acetylcholine (ACh). Ghayur et al. [47] demonstrated the potential inhibitory effect of ginger on cholinesterase well as its muscarinic activity and calcium antagonist action in rat stomach fundus tissue preparations. The authors studied the effects of an aqueous extract of dried ginger (Zo.Cr), consisting of terpenoids, flavonoids, secondary amines, phenols, alkaloids, and saponins, and also pure ginger compounds including 6-gingerol, 8-gingerol, 10-gingerol, and 6 -shogaol at the concentration of 1 mM. Stomach fundus was isolated from Sprague Dawley rats and demonstrate that treatment with Zo.Cr, showed a concentration-dependent spasmogenic effect, while Zo.Cr treatment led to a relaxation of the tissue. To confirm the spasmogenic effect of Zo.Cr, they used cholinergic antagonists such as hexamethonium (0.3 mM), atropine (0.1 mM) and methysergide (0.1 mM). Although the stimulating activity of Zo.Cr was resistant to both methysergide and hexamethonium treatment, the treatment with atropine has completely abolished the effect of Zo.Cr. Therefore, the spasmogenic effect of Zo.Cr was mediated by stimulation of muscarinic receptors. Hence, in the gastrointestinal tract atropine blocked the effect of nicotine (but not nicotine receptors) and also the increase of ACh mediated by muscarinic receptors. In order to evaluate if the spasmolytic activity of Zo.Cr and its pure compounds was due to a blocking of calcium channels, rat stomach tissues were treated with high concentrations of potassium (80 mM). Additionally to the effect on muscarinic receptors, Zo.Cr relaxed also the contractions induced by high potassium. Therefore, Zo.Cr exerted the action of the calcium antagonist compound. The increase of cytosolic calcium is linked to aging and mediated by calcium channel in the neuronal cell body. It can lead to the activation of apoptotic genes and to cholinergic neurons death, so calcium channel blockade compounds could be useful in preventing and mitigating AD. Moreover, the treatment with Zo.Cr in tissues induced with ACh (1 mM), inhibited cholinesterase activity. To confirm the results obtained, it was shown in vitro that Zo.Cr inhibited the enzymes AChE and BChe, preventing thus the cleavage of ACh into choline and acetate. Therefore, the double inhibitor effect of Zo.Cr could potentially be useful for AD treatment. The results were not replicated while using 6-, 8-, and 10-gingerol. Overall, the study showed a neuroprotective effect of ginger combining the activity of possible calcium antagonist as well as cholinesterase inhibitor.

The properties of ginger on memory deficits have also been evaluated by Sutalangka et al. in male Wistar rats. The animals underwent a bilateral intracerebroventricular injection of AF64A (2 nmol/2 μL, 2 μL/side), in order to induce cholinergic deficiency. The authors showed that oral administration of a combined extract of the analgesic and antibacterial *Cyperus rotundus* and *Zingiber officinale* (CP1), improved spatial memory in these animals assessed by Morris water maze test. Moreover, CP1 reduced the oxidative stress in the hippocampus, increasing CAT and SOD activity, and improved cholinergic functions by decreasing AChE activity. These effects were obtained independently by the doses of CP1 administered. Similarly, CP1 led to both an increase of neuronal density in the dentate gyrus and ERK1/2 levels, preserving the neuronal density in the hippocampus. Since the hippocampus is an important area related to memory, the administration of CP1 improved the memory deficits determined by hippocampal neurodegeneration. Therefore, the present study demonstrated that the efficacy of ginger, combined with other compounds, could be useful to counteract the memory deficits that characterize dementias like AD [48].

Kim et al. [49] demonstrated the neuroprotective effects of ginger on memory deficits. Male C57BL/6 mice orally received extracts of fresh ginger, dried ginger, or 6-gingerol as a pre-treatment. The model was then induced by an intraperitoneal injection of scopolamine (1 mg/kg) and behavioural changes were tested using Y-maze, Morris water maze, passive avoidance, and contextual fear conditioning tests. The groups pre-treated with ginger extracts gave better scores than the controls. Memory deficits induced by scopolamine were countered by the action of 6-gingerols via upregulation of brain-derived neurotrophic factor (BDNF), which has been shown to play an important role as trophic support in cholinergic neurons. Consequently, the lack of factors such as BDNF is critical for neuronal survival, synaptic plasticity, and cognitive processes. Therefore ginger, as well as its constituents such as 6-gingerol, have shown a therapeutic potential for the treatment and prevention of memory deficits.

The efficay of ginger root extract (GRE), a mixture composed of gingerol, shogaol and other plant biomarkers were studied by Zeng et al. in AD rats. Sprague-Dawley rats underwent a single intracerebroventricular injection of Aβ_1-40_ and continuous gavage of aluminum chloride for 4 weeks. After surgery, they were divided in four grups: GRE high-, medium-, and low-dose group and a group treated with huperzine A (100 mg/kg), an AChE inhibitor used as control. The high-dose group showed similar outcome as huperzine group after 35 days of treatment, while medium- and low-dose groups show no effects on behavioural dysfunctions. To assess how GRE can exhert its effects, immunohistochemical analyses were performed, revealed a comparable neuronal density between high-dose and huperzine group. A significative reduction of nuclear factor-kappa B (NF-κB), IL-1β and MDA levels suggested the anti-inflammatory action of GRE. Therefore, based on its neuroprotective properties and low toxicity, GRE can be considered potentially useful for preventing AD [50].

Additionally, the effects of ginger on behavioral and memory deficits were evalueted by Lim et al. [51] in APP/PSEN1 double-transgenic mice, an experimental model of AD characterized by the increase of aggregated Aβ and cognitive deficits. The authors reported that oral administration for 14 weeks of optimized combination of ginger and peony root (OCGP), inhibited the Aβ aggregation in the hippocampus evaluated by thioflavin T fluorescence assay and also prevented behavioral and memory deficits. Since the neuroinflammation is linked to amyloidogenesis, the expression levels of inflammatory markers including NF-κB, COX-2, TNF-α, IL-1β, and IL-6 as well as the activation of astrocytes in the brain was evaluated. Noteworthy, that OCGP treatment reduced the levels of COX-2 and glial fibrillary acid protein (GFAP), expressed in astrocytes.

Moon et al. demonstrated the role of 6-shogaol in memory deficits, acting on astrocytes and microglia activation. The authors induced the model by a unilateral hippocampal injection of Aβ_1-42_ oligomers (3 μL, 10 μM), using saline solution for sham-treatment. Mice were divided in four groups according to their treatment: saline solution, 6-shogaol, and donepezil (2 mg/kg per day), while the last group was the sham-mice. The results of study reported that 6-shogaol treatment was able to reduce the inflammation and neuronal death, ameliorating in such way the learning and mitigating the memory deficits. 6-shogaol has significantly reduced the activation of astrocytes and microglia in the hippocampus, as shown by the reduction of GFAP and macrophage-1 antigen (Mac-1) expression levels [52].

Also Na et al. studied in vitro and in vivo the neuroprotective effects of 6-shogaol in AD. In particular, the authors investigated the effects of 6-shogaol on the cysteinyl leukotriene 1 receptor (CysLT1R) and cathepsin B both involved in the pathogenesis of AD. CysLT1R is a receptor activated by the inflammatory mediator cysteinyl leukotrienes (CysLTs), which is produced by 5-lipoxygenase (5-LO) during the arachidonic acid (AA) metabolic pathway. CysLT1R is expressed in glial cells and neurons after a traumatic injury, as well as cathepsin B, known to be involved in neuronal death and consequently in behavioural deficits. The authors observed that Aβ_1–42_ can lead to neurodegeneration mediate by cathepsin B, activated after expression of CysLT1R. Conversely, inhibition of cathepsin B can reduce Aβ production. In vitro results suggested that pre-treatment with 6-shogaol 1 h prior induction of hippocampal HT22 cells mice with Aβ_1–42_, led to an increase in cell viability compared to Aβ_1–42_ (5 μM) treated controls cells. Additionally, lactate dehydrogenase assay revealed that 6-shogaol can inhibit cytotoxicity induced by Aβ_1–42_. Furthermore, it was shown a significant improvement in behavioural and memory tests when APP/PSEN1 mice orally received 6-shogaol for two months. Hence, the study results suggested that 6-shogaol acting as a CysLT1R/cathepsin B inhibitor could act as neuroprotector, preventing neuronal death and cognitive deficits [53].

Moreover, Na et al. [54] showed both in vitro and in vivo the neuroprotective effects of 6-shogaol in the activation of Sortilin-related receptor 1 (SORL1), a neuronal shunting protein involved in the processing of APP and secretion of Aβ. The in vitro study demonstrated that treatment with 6-shogaol suppressed Aβ aggregation in HT22 cells induced with SORL1 small interfering RNA (siRNA) (10 mM). The presence of SORL1 siRNA in HT22 cells inhibited SORL1 and led to the increase in expression of beta-site amyloid precursor protein cleaving enzyme 1 (BACE1), involved in the formation of Aβ monomers. By contrast, treatment with 10 mM 6-shogaol exerted a neuroprotective effect through the activation of SORL1 and the significant reduction of BACE and Aβ. To confirm the in vitro results, the authors performed the study in APP/PSEN1 mice administered orally with 6-shogaol. In vivo study results also confirmed that 6-shogaol inhibited Aβ aggregation in the animal brains, so 6-shogaol could be a potential useful compound for treating AD.

Huh et al. [55] demonstrated the neuroprotective effects of fermented ginger enriched with 6-paradol. ICR male mice underwent a single intraperitoneal injection of scopolamine (1.1 mg/kg) to induce the model, then they were treated orally with ginger enriched with 6-paradol or unfermented ginger. Donepezil hydrochloride was used as the control. 6-paradol showed to enhance the neuroprotective effects of ginger, evaluated by recognition memory compared to control mice treated with unfermented ginger. Additionally, to demonstrate the neuroprotective effects of ginger in AD, mice were unilaterally injected into the hippocampus with Aβ_1–42_ (1 mg/ml) and fermented ginger was administered orally at different concentrations. The study results reported that fermented ginger improved memory deficits by protecting hippocampal neurons from neurodegeneration induced by Aβ_1–42_.

Jafarian et al. in vivo showed the neuroprotective effects of zerumbone. To induce the experimental model characterized by memory deficits, hyperactivity, anxiety-like behaviours, and locomotor activities, Sprague-Dawley rats were injected intraperitoneally with scopolamine (20 mg/kg). Intraperitoneal administration of zerumbone in a single dose improved learning and prevented memory deficits, reduced hyperactivity and anxiety-like behaviours, evaluated through open field tests, elevated plus maze and Morris water maze tests. Most likely, the parameters related to the evaluation of anxiety could involve anxiolytic or sedative processes mediated by gamma-aminobutyric acid (GABA) A receptors. Therefore, compounds such as zerumbone could potentially be useful not only to improve the memory and learning, but also to reduce the anxiety, ameliorating the quality of life of the patients [56].

Additionally, the effects of zerumbone were studied by Li et al. [57] both in vivo and in vitro. In the in vivo experiment, transgenic APP/PSEN1 mice received daily gavage of zerumbone for 20 days. The treatment improved the memory deficits, learning and nesting ability, and also social interaction. Moreover, in these animals zerumbone has significantly reduced Aβ deposition and neuroinflammation both in the hippocampus and in the cortex, avoiding Aβ aggregation and thus contributing to the improvement of the cognitive and non-cognitive deficits that characterize the AD. Furthermore, the authors evaluated the effects of zerumbone in N9 microglial cells and primary microglial cells isolated from the cortex of C57BL/6J mice. To induce the in vitro experimental model, microglial cells were stimulated with lipopolysaccharide (1 μg/ml) or Aβ_1–42_ (10 μM), and incubated for 24 h with or without zerumbone. Treatment with zerumbone, in addition to suppressing neuroinflammation, favoured the passage of microglial cells from a pro-inflammatory to an anti-inflammatory phenotype, through the involvement of MAPK (mitogen-activated protein kinase)/NF-κB signalling. Indeed, the administration of zerumbone into microglial cells attenuated the activation of p38 MAPK/NF-κB induced by Aβ. As confirmation, it has been shown that zerumbone, suppressed the expression of Prostaglandin E2 (PGE2), as well as significantly reduced the phosphorylated forms of ERK1/2 and p38 MAPK, and also inhibit the translocation of p65 NF-κB into the nucleus, thus exerting its neuroprotective effects. Therefore, the study suggested an important action of zerumbone that, through inhibition of MAPK signalling, can promote the anti-inflammatory microglial polarization.

Kim et al. [58] investigated the anti-inflammatory and antioxidant effects of zingerone which, through the modulation of NF-kB and reactive oxygen species (ROS) production, could potentially be useful for the treatment of aging-related diseases. The authors used YPEN-1 rat prostate endothelial cells pre-treated with zingerone for 2 h and rat kidney cells obtained from aged male Fischer 344 rats, fed with zingerone for 10 days. The results of the study showed the inhibitory effect of zingerone on NF-kB in YPEN-1 cells transfected with pNF-κB-Luc vector, and evaluated through the reduction of luciferase activity. Moreover, it was confirmed that zingerone could inhibit nuclear translocation of NF-kB and the transcription of pro-inflammatory mediators, such as COX-2 and inducible nitric oxide synthetase (iNOS), through the downregulation of aging-related pathways including MAPK, ERK, p38, and JNK. Zingerone proved to act as a scavenger and to possess anti-inflammatory properties, so it can be useful to slow down the aging process.

The neuroprotective effects of ginger on neuronal death induced by hydrogen peroxide (20–50 μM), aluminum (50–200 μM), zinc (10–50 μM), or tamoxifen (0.1–0.5 μM), an antagonist of estrogen receptor (ER), were also shown in vitro by Mizuno et al. [59]. To perform the study, the authors used GT1–7 cells (immortalized hypothalamic neurons), a model widely used to assess neurotoxicity and endocrine disruption. The results of the study showed that 25 ppm ginger essential oil as well as other essential oils including damask rose, eucalyptus, fennel, geranium, kabosu, mandarin, myrrh and neroli reduced peroxide-induced neurotoxicity of hydrogen in GT1–7 cells. Instead, ginger essential oil and geranium essential oil enhanced the neurotoxicity of tamoxifen in a dose-dependent manner. Although the molecular mechanism is still under study, essential oils may act as either estrogen receptor (ER) agonists or antagonists, to regulate the mechanism of tamoxifen-induced cell death.

To prevent neuronal loss in age-related neurodegenerative disorders, Lee et al. [60] demonstrated the effects of [6]-gingerol in SH-SY5Y cells Aβ_25–35_ (2.5, 7.5, 20 µM) induced. [6]-gingerol pre-treatment improved cell viability in a concentration dependent manner, as shown by the reduction of cytotoxicity induced by Aβ_25–35_. Furthermore, [6]-gingerol prevented apoptosis by avoiding both DNA fragmentation and reduction of mitochondrial membrane potential. Confirmation of anti-apoptotic effects of [6]-gingerol was shown by reverting the effects of Aβ_25–35_ on B-cell lymphoma 2 (Bcl-2)/BCL2-associated X apoptosis regulator (BAX) ratio. Moreover, the authors demonstrated the antioxidant action of [6]-gingerol on the increase of antioxidant enzymes c-glutamylcysteine ligase (GCL) and Heme Oxygenase-1 (HO-1), most likely due to nuclear factor erythroid 2-related factor 2 (NRF2). Overall, the results of the study suggested that anti-apoptotic action of [6]-gingerol could be useful to preventing neurodegeneration.

The neuroprotective effects of 6-gingerol were shown by Zeng et al. [61] in rat pheochromocytoma PC12 cells. In order to evaluate the cell viability, as well as the anti-apoptotic effects of 6-gingerol, before to induce the experimental model with Aβ_1–42_ (10 μM), the PC12 cells were pre-treated with 6-gingerol. The results showed that 6-gingerol significantly decreased the oxidative stress, evaluated by reduction of ROS levels, MDA and nitric oxide (NO) production, as well as by the increase of SOD activity compared to the control group. Furthermore, the neuroprotective role of 6-gingerol has also been demonstrated through the activation of AKT and inhibition of GSK-3. GSK-3 inhibition can reduce APP cleavage, likely through an NF-κB-mediated mechanism, avoiding Aβ formation.

Kim et al. [62] investigated both in PC12 cells induced with Aβ_1–42_ (1.0 g/mL) and primary neuronal cells treated with Aβ_1–42_ (3.0 g/mL), the neuroprotective effects of 27 herbs as a potential treatment for AD. Even if most herbs extracted with aqueous methanol and chloroform reported cytotoxicity at high concentrations or no beneficial effect, seven herbs including ginger extract led to an increase in the cell viability. Overall, the results of the study once again demonstrated the neuroprotective role of ginger in AD.

In addition, Mathew et al. [63] demonstrated in primary hippocampal cells of adult rats the neuroprotective effects of ginger. After pre-treatment with dry ginger, the experimental model was induced with monomeric Aβ (200 μM). The results of the study showed that ginger extract improved cell survival by reducing the Aβ-neurotoxicity. Moreover, ginger pre-treatment prevented both aggregation and dissociation of preformed Aβ oligomers.

Guo et al. [64] developed assays to identify compounds able to block Aβ aggregates. The neuroprotective role of ginger was demonstrated by using the microplate assay useful to calculate the concentration to be used to obtain the desired effect, as well as the tissue assay used to give an indication of how the molecules could act in vivo. Indeed, it was demonstrated, in brain tissue from AD transgenic mice, that aggregation of Aβ was prevented by pre-treatment with ginger extracts.

Another in vitro study performed by Grzanna et al. [65] demonstrated the anti-inflammatory effects of ginger in human monocytic THP-1 cells. A solution containing ginger extracts (GE), composed by *Zingiber officinale* Roscoe and *Alpinia galangal,* was used as pre-treatment, then the cells were exposed to different inflammatory mediators including TNF-α, IL-1β, lipopolysaccharide (LPS) and also Aβ_1–42_. GE-treatment, inhibited expression levels of proinflammatory mediators such as TNF-α, IL-1β, COX-2, and also chemokines monocyte chemoattractant protein-1 (MCP-1)/C-C motif chemokine ligand 2 (CCL2), macrophage inflammatory protein-1α (MIP-1α)/C-C motif chemokine ligand 3 (CCL3), and CXC ligand 10 compared to controls cells. Consequently, the study reported the efficacy of GE in preventing the inflammatory cascade most likely according to a mechanism of action different by NSAIDs, as well as the likely involvement of NF-kB as possible inflammatory molecular target of ginger.

The anti-inflammatory effects of ginger and its bioactive compounds by NF-κB modulation were demonstrated by Ho et al. [66] in THP-1 cells lipopolysaccharide (LPS) and adenosine 5’-triphosphate (ATP) induced. In THP-1 cells the pre-treatment with shogaols /gingerols including 10-gingerol, 6-shogaol, 8-shogaol, 10-shogaol, inhibited both the secretion of IL-1β and activation of nucleotide-binding domain and leucine-rich repeat-containing family, pyrin domain-containing 3 (NLRP3) inflammosome regulates by NF-κB. Additionally, it was shown that ginger compounds reduced the expression levels of caspase-1, induced by ATP activation. Shogaols demonstrated a greater inhibitory power on the inflammatory pathway. It was also found that the inhibitory action of shogaols on IL-1β and NLRP3 was related to the presence of α, β-unsaturated carbonyl groups. Therefore, an increase in the length of alkyl chain in shogaols reduced both the inhibitory action on IL-1β and the anti-inflammatory, antiproliferative, and antioxidant effects. The increase of the alkyl side chain in gingerols most likely induced an increase in the capacity of these compounds to inhibit the secretion of IL-1β mediated by NLRP3 inflammasome, thus also leading to an improved anti-inflammatory effect. Given its role as a modulator of immune responses and its aberrant activation in the pathogenesis of inflammatory diseases, studying compounds that target NLRP3 inflammasome such as ginger could be useful to counteract the chronic inflammation that common in dementias.

Additionally, Chen et al. [67] in bone marrow-derived macrophages from C57BL/6J mice demonstrated the anti-inflammatory effects of ginger. The authors, before inducing the experimental model with LPS and ATP, pretreated the cells with exosome-like nanoparticles (ELNs) of ginger rhizomes (G-ELNs), easily absorbed by macrophages, and containing protein, lipid, and RNA. The results of the studies confirmed the anti-inflammatory effects of ginger. Indeed, G-ELNs treatment inhibited the assembly of the NLRP3 inflammasome, blocking apoptotic speck protein containing a caspase recruitment domain (ASC), involved in the recruitment of caspase1, secretion and release of proinflammatory cytokines IL-1β and IL-18 and in pyrooptosis.

**Figure 4:**
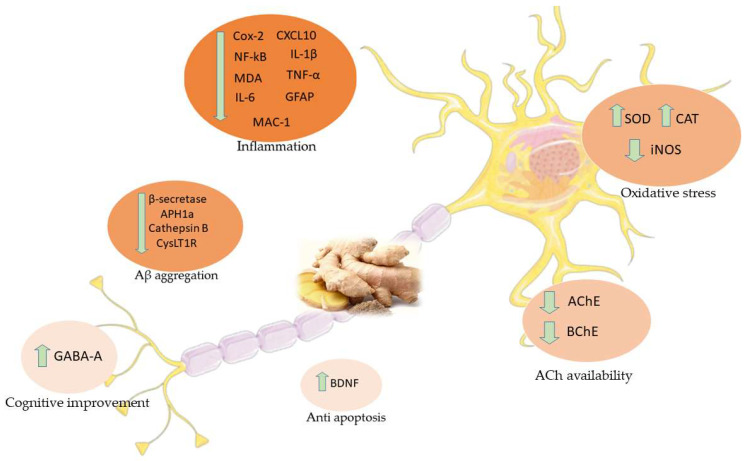
Main effects of ginger in AD studies. COX-2: Cyclooxygenase 2; CXCL10: C-X-C Motif Chemokine Ligand 10; NF-κB: Nuclear factor kappa-B; IL-1β: Interleukin 1-beta; MDA: Malondialdehyde; TNF-α: Tumor necrosis factor-α; IL-6: Interleukin 6; GFAP: Glial fibrillary acid protein; MAC-1: Macrophage-1 antigen; SOD: Superoxide dismutase; CAT: Catalase; iNOS: Inducible nitric oxide synthetase; APH1a: Gamma-secretase subunit APH-1A; CysLT1R: Cysteinyl leukotriene 1 receptor; AchE: Acetylcholinesterase; BChE: Butyrylcholinesterase; BDNF: Brain-derived neurotrophic factor; GABA-A: Gamma-Aminobutyric acid-A.

According to the evidences examined so far (Table 1), ginger and its compounds proved to exert different beneficial effects in AD models (Figure 4).

## 6. Vascular Dementia

VD is one of the most common type of dementia, second only to AD [7]. As people live longer and diagnostic tools became more efficient, so the terminology has to evolve: patients who showed the onset and not yet severe form of AD are grouped under the Mild Cognitive Impairment term (MCI) [68]. While patients suffering from cognitive deficit after cerebrovascular disease belong to the Vascular Mild Cognitive Impairment group (VaMCI) [69]. More general, in Vascular Cognitive Impairment (VCI) comprise all the syndromes where there is evidence of cognitive impairment after vascular brain injury. However, it is important to point out that VD and AD are not diseases completely separated from each other: the BBB breakdown plays an important role in the main forms of dementias, so vascular dysfunctions contribute also in other diseases than VD [70]. In ancient times, dementias were thought to be due to the “hardening of arteries”, so the vascular system was always recognized as a relevant player in dementias onset [71].

An important distinctive feature of brain vascular system is the BBB: it is a complex association between endothelial cells, pericytes, and astrocytes, with the function of protecting the brain from the entry of unwanted molecules, substances, or organisms. In further detail, the access to the brain is very limited to ions and, of course, pathogens and toxins. Water-soluble substances cannot cross the BBB and this represents an obstacle for drug delivery [72]. The efficiency of this barrier is guaranteed at different levels. Tight and adherents junctions between endothelial cells restrict the transport of molecules between brain interstitial space and blood-flow [73]. Since the brain requires 20% of the blood supply of all the body, it is no surprise that any accident to the vascular system can have dramatic consequences, and the cognitive impairment is only one of them. Even if it has been difficult to clarify how every single condition contributes to the VD, some general conclusions can be assessed. The diminished blood flow in the brain region plays an important role in the development of dementia, since it correlates with a decrease in brain activity and cognitive function, even if this condition is reversible [74]. It is important to mention that the timing is crucial, because a long deprivation of blood flow, resulting in oxygen and glucose deprivation, leads to neuronal death and this condition is not reversible [75]. In addition, oxidative stress and inflammation, both very common in the elderly population, play a key role in neurovascular dysfunction. Oxidative stress and inflammation strictly correlate and one reinforce the other, because free radicals activate pro-inflammatory transcription factors and inflammation decrease the efficiency of antioxidant defence mechanism of the cells. Endothelial cells subjects to oxidative stress secrete prostanoid and vascular endothelial growth factors, promoting vascular leakage [76]. Oxidative stress and inflammation also have a role in blocking the differentiation and proliferation of oligodendrocytes, thus impeding the re-myelination of damaged white matter, and they can cause the death of BBB cells [77,78]. BBB disruption permit the access into the brain not only to blood cells, but also to pathogens and toxins, as mentioned before, and a damaged area of BBB can transfer the blood-derived substances also in areas where the barrier is still intact [79].

To date, unfortunately, there are no real treatments to cure vascular dementia, while we try to reduce its progression. Therefore, the use of phytocompounds such as ginger alone or in combination with other compounds could potentially be useful for the VD treatment.

## 7. Effects of Ginger in VD Studies

Several experimental studies have shown the neuroprotective effects of ginger as a potential treatment for VD.

Dementia following cerebral ischemia in metabolic syndrome (MetS) is often severe and currently there are no effective treatments. Therefore, finding therapeutic strategies capable of reducing dementia and memory deficits induced by ischemic stroke in the MetS is necessary.

Wattanathorn et al. [80] studied the combined effects of mulberry fruit and ginger (PMG) contained in a phytosome in an in vivo experimental model of MetS. To induce MetS, male Wistar rats underwent a high-fat and carb diet for 16 weeks. The ischemic injury was modelled via occlusion of the right middle cerebral artery (Rt. MCAO), obtained by isolation of right external and internal carotid artery with nylon. Before and after surgery rats were treated orally with PMG. Since inflammation and oxidative stress are important to inducing cognitive deficits, the authors evaluated the levels of AChE, MDA, neuronal density, SOD, glutathione peroxidase (GPx), IL-6 in the cerebral cortex and hippocampus and the possible underlying mechanisms of signal transduction via ERK pathway. The results showed that the administration of PMG improved the cognitive deficits and also the neuronal density both in the cerebral cortex and hippocampus. Additionally, PMG treatment reduced the activity of AChE, MDA and also the expression level of IL-6, as well as led to SOD increase, catalase (CAT), GPx and ERK phosphorylation both in the cortex and hippocampus. Most likely, PMG can improve memory deficits by reducing the level of AChE which in turn can stimulate ERK phosphorylation to suppress inflammation. Therefore, PMG, leads to the increase of ACh availability and the stimulation of ERK by reducing the level of AChE, suppressing inflammation and thus improving cognitive deficits.

Additionally, Palachai et al. [81] investigated the neuroprotective effects of PMG in MetS male Wistar rats fed with high-fat and carbs diet. Rats then underwent ischemic damage/cerebral reperfusion using Rt. MCAO. To evaluate the neuroprotective effect of PMG on memory deficits, PMG was administered orally. As in the previous study, ischemic damage favoured the increase in oxidative stress and inflammation in the brain, which in turn led to impairment of ATPase pumps, especially Na^+^ and K^+^, ATPase triggers brain edema and influenced neurological deficits. The administration of PMG led to the reduction of inflammation and oxidative stress evaluated by decreased levels of NF-κB, TNFα, and MDA. The treatment also leads to an increase in the activity of SOD, CAT, and GPx and also increased the expression of Peroxisome proliferator-activated receptor γ (PPARγ). Indeed, both oxidative stress and inflammation can be influenced by the expression of PPARγ. In this regard, it has been shown that PPARγ agonists could exert a neuroprotective role against cerebral ischemia by suppressing oxidative stress and inflammation. Overall, the study results reported that PMG could be a potential candidate to protect against brain damage and MCAO in MetS conditions.

Another study performed by Wattanathorn et al. [82] showed the neuroprotective effect of Ginger in rats with focal cerebral ischemia. In order to evaluate the effects on cognitive function, before and after inducing the Rt. MCAO model, ginger rhizome was administered orally to male Wistar rats. The results of the study showed that extract of ginger rhizome improved cognitive functions, assessed by the Morris Water Maze test, and also reduced the oxidative stress. Moreover, ginger has been shown to reduce the volume of cerebral infarction and improve neuronal density. Thus, ginger provided a neuroprotective effect by increasing neuronal density in the hippocampus, improved spatial memory, and reduced cerebral infarction volume. Although more studies are needed, ginger could be a potential candidate for countering focal cerebral ischemia.

The neuroprotective effects of *Houshiheisan*, an herbal blend composed by chrysanthemun flower, divaricate saposhnikovia root, Manchurian wild ginger, cassia twig, Szechwan lovage rhizome, platycodon root, ginseng, Chinese angelica, large-head atractylodes rhizome, Indian bread, and zingiber and used as classic prescription for stroke in traditional Chinese medicine, were evaluated by Wang et al. [83] in a rat model of focal cerebral ischemia. Before and after the surgery, the authors gavaged the animals with *Houshiheisan*. The results of the study showed that treatment with *Houshiheisan* reduced damage of the ischemic penumbra, the area between the undamaged region and the core. Moreover, *Houshiheisan* treatment protected the neurovascular units from abnormal deposition of Aβ, thus favouring the stabilization of the neurovascular units and reducing their injuries. Overall, the results of the study demonstrated that *Houshiheisan*, through the combined action of its compounds, could be useful for protecting neurovascular units after cerebral ischemia.

The neuroprotective effects of 6-paradol on focal cerebral ischemia and inflammation were studied by Gaire et al. [84] both in vivo and in vitro. Among 5 paradol derivatives such as 2-, 4-, 6-, 8- and 10-paradol, 6-paradol was selected as the compound with the greatest anti-inflammatory effect. Since one of the pathological characteristics of cerebral ischemia is represented by the neuroinflammation following microglia activation, it is necessary to find useful therapeutic strategies to reduce the neuroinflammatory responses in activated microglia. To study the effects of 6-paradol, Gaire et al. [84] used MCAO/reperfusion (M/R) mice administered orally immediately after reperfusion with 6-paradol. The results of the in vivo study showed that 6-paradol reduced brain damage, improved neurological deficits evaluated by motor and sensory function, and also improved neuronal survival. Moreover, 6-paradol reduced the evaluated microglial activation through the reduction of TNF-α and iNOS. In murine microglial BV-2 cells pre-treated with 6-paradol and stimulated with LPS (100 ng/mL) for 24 h, it was observed the reduction of neuroinflammation following microglial activation as shown by the reduction of iNOS and proinflammatory cytokines IL-6 and TNF-α [84].

**Figure 5:**
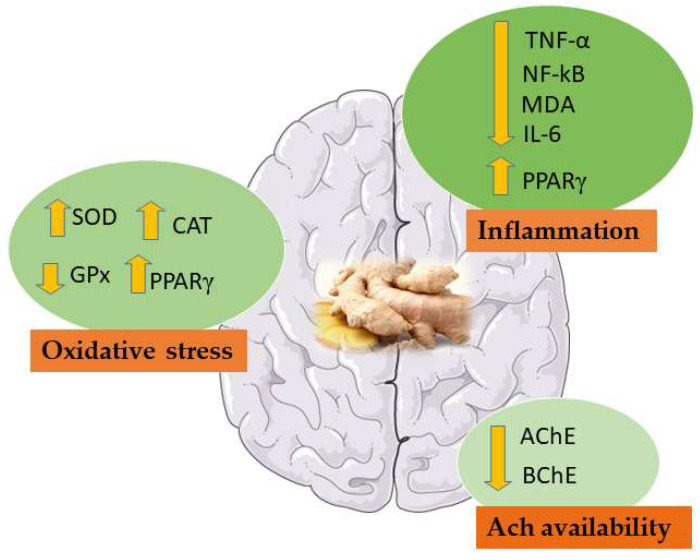
Main effects of ginger in VD studies. NF-κB: Nuclear factor kappa-B; MDA: Malondialdehyde; TNF-α: Tumor necrosis factor-α; IL-6: Interleukin 6; SOD: Superoxide dismutase; CAT: Catalase; AChE: Acetylcholinesterase; BChE: Butyrylcholinesterase; GPx: glutathione peroxidase; PPARγ: peroxisome proliferation activator receptor γ.

According to the experimental studies analysed so far (Table 2), ginger and its derivate can be considered as potential treatment in VD (Figure 5).

## 8. Conclusions

The appeal of natural compounds has increased in the last decades and the scientific community seems to regain interest in them, in order to avoid the side effects of synthetic drugs or to increase their efficacy. Ginger has proven to be a valuable candidate for the treatment of dementia, with evidence also supporting its use in prevention. Several experiments have enlightened ginger’s effect in hampering multiple phases of dementia developing, from neurodegeneration to neuroinflammation, supporting on the other hand the survival rate of neurons. Even if at present ginger does not represent a definitive cure for advanced stages of dementia, further studies could discover more beneficial properties, and the refinement of dosage, route, and timing of administration could represent a valid support for current therapies.

## Figures and Tables

**Table 1:** Summary of reported studies on ginger effects in AD treatment of prevention.

Experimental Model	Compound	Route/Dose	Effects	Reference
Molecular docking	Ginger compounds	.	Evaluation of molecular targets based on the measure of the binding energy is AChE.	[41]
Molecular docking	Active compounds of ginger extracts	.	Inhibition of AChE activity by the Trp86 choline binding site.	[42]
Molecular docking	Ginger compounds	.	Inhibition of BChE activity.	[43]
In vitro target enzyme assays, silico docking and simulation of absorption, distribution, metabolism, excretion, and toxicity	Zerumbone	.	Reduction of AChE and BChE activity.	[44]
Computational model: koniocortex	Terpenoid-based nutrients	.	Expression of GABA-A receptor and improve cognitive function.	[45]
Male Swiss albino mice	Gingerol	Intraperitoneal 10 and 20 mg/kg	Reduction of the levels of Aβ_42_, β-secretase, APH1a and COX-2. Increased of α-secretase activity. No behavioural and memory deficits. Probable improvement of neuroinflammation and amyloidogenesis.	[46]
Sprague Dawley rats stomach	Zo.Cr	0.03–5.00 mg/mL	Possible calcium antagonism and cholinesterase inhibition.	[47]
Male Wistar rats	Extract of *Cyperus rotundus* and *Zingiber officinale* (CP1)	Gavage 100, 200 and 300 mg/kg	Oxidative stress reduction in hippocampus. Improved cholinergic function. Improved memory deficit.	[48]
Male C57BL/6 mice	fresh ginger, dried ginger, 6-gingerol	Gavage fresh ginger 100 or 200 mg/kg dried ginger 100 or 200 mg/kg 6-gingerol 10 or 25 mg/kg	Upregulation of BDNF. Prevention of memory deficits.	[49]
Sprague-Dawley rats	Ginger root extract (GRE)	Intragastric GRE high-dose group 4 gr/kg, GRE medium-dose group 2 gr/kg, GRE low-dose group 1 gr/kg huperzine A group 100 mg/kg	Reduction of inflammatory markers (high-dose group). Improved behavioural tests (high-dose group).	[50]
Male APP/PSEN1 mice	Ginger and peony root (OCGP)	Gavage 50 or 100 mg/kg/day 14 weeks	Aβ aggregation inhibition in hippocampus. Behavioural and memory deficits prevention. Reduction of inflammatory marker expression. Reduction of astrocytes activation.	[51]
Male ICR mice	6-shogaol	Gavage 10 mg/kg/day	Reduced astrocytes and microglia activation. Inflammation and neuronal death reduction. Improvement in learning and memory.	[52]
APP/PSEN1 mice and HT22 cells	6-shogaol	Gavage 5 and 20 mg/kg and pre-treatment 10 μM	Improving of behavioural and memory deficits (in vivo). Increased cell availability (in vitro). Inhibition of cytotoxicity Aβ_1–42_-induced (in vitro).	[53]
HT22 cells and APP/PSEN1 mice	6-shogaol	1, 5, 10 and 20 mM Gavage 5 or 20 mg/kg every 2 days for 2 months	Aβ aggregation suppression.	[54]
Male ICR mice	Ginger + 6-paradol unfermented ginger fermented ginger	Gavage ginger + 6-paradol 100 mg/kg unfermented ginger 100 mg/kg fermented ginger 50,100,200 mg/kg per day	Neuroprotective effect (ginger + 6-paradol). Protection to neurodegeneration in hippocampus (fermented ginger). Improved memory deficits (fermented ginger).	[55]
Sprague-Dawley rats	Zerumbone	Intraperitoneal 1 and 10 mg/kg	Improved the memory deficits. Ameliorate anxiety-like behaviours.	[56]
Transgenic APP/PSEN1 mice and N9 microglial cells and primary microglial cells from C57BL/6J mice	Zerumbone	Gavage 25 mg/kg and 1, 3 o 10 μg/ml	Improved memory, learning, social interaction, and nesting (in vivo). Reduced Aβ aggregate formation and neuroinflammation in hippocampus and cortex (in vivo). Neuroinflammation suppression (in vitro).	[57]
YPEN-1 rat prostate endothelial cells and rat kidney cells from male Fischer 344 rats	Zingerone	YPEN-1 cells pre-treated 1, 5, 10, or 20 μM and rat kidney cells obtained from male rats fed with zingerone 2 or 8 mg/kg/day for 10 days	Reduction in inflammation and oxidative stress.	[58]
GT1–7 cells	Ginger essential oil	25 ppm	Reduction of peroxide-induced neurotoxicity. Enhanced tamoxifen neurotoxicity.	[59]
SH-SY5Y cells	[6]-gingerol	Pre-treatment 3, 10 μM	Improved cell viability. Reduction Aβ_25–35_ citotoxicity. Oxidative stress reduction.	[60]
PC12 cells	6-gingerol	Pre-treatment 40, 80, 120, 200, and 300 μM	Oxidative stress reduction. Decreased Tau hyperphosphorylation. Decreased Aβ formation.	[61]
PC12 cells and primary neuronal cells	Ginger extract	20 g/mL	Improved cell viability.	[62]
Rat primary hippocampal cells	Dry ginger	0.02, 0.1, and 0.2 mg in 2 μL of DMSO	Prevention of Aβ-aggregation. Neurotoxicity reduction. Improved cell survival.	[63]
Brain tissue from AD transgenic mice	Ginger extract	Dilutions (1:20, 1:40 and 1:100)	Aggregation of Aβ prevention.	[64]
Human monocytic THP-1 cells	Ginger extract composed by *Zingiber officinale* and *Alpinia galangal*	Pre-treatment 255 mg	Reduction of inflammation.	[65]
THP-1 cells	Shogaols/gingerols	Pre-treatment 20 μM	Reduction of inflammation.	[66]
Bone marrow-derived macrophages	Exosome-like nanoparticles of ginger rhizomes	/	Inhibition of inflammasome formation.	[67]

Aβ: β-amyloid; Zo.Cr: dried ginger; AD: Alzheimer’s disease; BDNF: brain-derived neurotrophic factor; GRE: ginger root extract; APP / PSEN1: amyloid precursor protein/presenilin 1; GE: ginger extract.

**Table 2:** Summary of reported studies on ginger effects in VD treatment of prevention.

Model	Compound	Route, Dose, Timing	Results	Reference
Male Wistar rats Rt. MCAO	PMG	Gavage. 50, 100, 200 mg/kg 21 days	Improved cognitive deficits. Reduced AChE activity. Decreased inflammation markers. Increased antioxidant levels.	[80]
Male Wistar Rats Rt. MCAO	PMG	Gavage. 50, 100, 200 mg/kg 21 days	Improved cerebral ischemic damage. Improved cerebral edema. Decreased neurological deficits. Inflammation and oxidative stress reduction.	[81]
Male Wistar Rats Rt. MCAO	Ginger rhizome	Gavage. 200 mg/kg Before and after surgery	Improved cognitive function. Reduced oxidative stress. Increased hippocampal neuron density. Reduced cerebral infarction volume.	[82]
Rat Focal ischemia	*Houshiheisan*	Gavage. 2.59, 7.7 and 10.5 g/kg Before and after surgery.	Reduced damage to the ischemic penumbra. Vascular protection from Aβ deposition. Stabilization of neurovascular unit.	[83]
Mice MCAO/reperfusion and BV2 cells LPS stimulation 100 ng/mL	6-paradol	Gavage. 1, 5, 10 mg/kg after reperfusion and Pre-treatment 10 ug/mL	Reduced microglia activation. Reduced brain damage. Improved motor and sensory function. Neuroinflammation reduction. Reduction of iNOS. Reduction pro-inflammatory cytokines	[84]

VD: vascular dementia; PMG: mulberry fruit and ginger; Rt. MCAO: right middle cerebral artery occlusion; Aβ: β-amyloid; LPS: lipopolysaccharide; iNOS: nitric oxide synthase.

## Data Availability

No new data were created or analysed in this study. Data sharing is not applicable to this article.

## Abbreviations

DSM-5	Diagnostic and Statistical Manual of Mental Disorders
AD	Alzheimer’s Disease
VD	Vascular Dementia
NFTs	Neurofibrillary tangles
Aβ	Beta-amyloid
BBB	Blood-brain barrier
COX-1	Cyclooxygenase 1
APOE	Apolipoprotein E
PSEN 1	Presenilin 1
PSEN 2	Presenilin 2
APP	Amyloid precursor protein
GSK-3	Glycogen synthase kinase-3
ROS	Reactive oxygen species reactive
MDA	Malondialdehyde
NSAIDs	Non-steroidal anti-inflammatory drugs
COX-2	Cyclooxygenase-2
5-LOX	5-lipoxygenase
LTs	Leukotrienes
SAD	Late-onset sporadic Alzheimer Disease
FAD	Early-onset familial Alzheimer Disease
APH1a	Gamma-secretase subunit APH-1A
AChE	Acetylcholinesterase
ACh	Acetylcholine
Zo.Cr	Dried ginger
BChE	Butyrylcholinesterase
SOD	Superoxide dismutase
CAT	Catalase
ERK	eEtracellular signal-regulated kinase
BDNF	Brain-derived neurotrophic factor
GRE	Ginger root extract
NF-κB	Nuclear factor kappa-B
IL	Interleukin
GFAP	Glial fibrillary acid protein
Mac-1	Macrophage-1 antigen
CysLT1R	Cysteinyl leukotriene 1 receptor
CysLTs	Cysteinyl leukotrienes
AA	Arachidonic acid
SORL1	Sortilin-related receptor 1
siRNA	Small interfering RNA
BACE1	Beta-site amyloid precursor protein cleaving enzyme 1
GABA	Gamma-Aminobutyric acid
MAPK	Mitogen-activated protein kinase
PGE2	Prostaglandin E2
iNOS	Inducible nitric oxide synthetase
JNK	C-jun N-terminal kinase
ER	Estrogen receptor
Bcl-2	B-cell lymphoma 2
BAX	BCL2-associated X: apoptosis regulator
NRF2	Nuclear factor erythroid 2-related factor 2
GCL	C-glutamylcysteine ligase
HO-1	Heme Oxygenase-1
NO	Nitric oxide
GE	Ginger extracts
LPS	Lipopolysaccharide
TNF-α	Tumour necrosis factor-α
MCP-1	Monocyte chemoattractant protein-1
CCL2	C-C motif chemokine ligand 2
MIP-1α	Macrophage inflammatory protein-1α
CCL3	C-C motif chemokine ligand 3
ATP	Adenosine 5’-triphosphate
NLRP3	Nucleotide-binding domain and leucine-rich repeat-containing family, pyrin do-main-containing 3
ELNs	Exosome-like nanoparticles
G-ELNs	Ginger rhizomes
ASC	Apoptotic speck protein containing a caspase recruitment domain
TACE	TNF-α converting enzyme
NMDA	N-methyl-D-aspartate
Mol1	[(E)-1,7-bis (4-hydroxy-3-methoxyphenyl) hept-4-en-3-on]
Mol2	[1-(3,4-dihydroxy-5-methoxyphenyl)-7-(4-hydroxy-3-ethoxyphenyl) heptane-3,5-diyl diacetate]
G3	5-[(2S,4R,6R)-4-hydroxy-6-[2-(4-methoxyphenyl)ethyl]oxan-2-yl]-3-methoxybenzene-1,2-diol
PPARγ	Peroxisome proliferation activator receptor γ
MCI	Mild Cognitive Impairment
VaMCI	Vascular Mild Cognitive Impairment
VCI	Vascular Cognitive Impairment
MetS	Metabolic syndrome
PMG	Mulberry fruit and ginger
Rt. MCAO	Right middle cerebral artery
GPx	Glutathione peroxidase
